# The impact of frailty in aortic valve surgery

**DOI:** 10.1186/s12877-020-01716-3

**Published:** 2020-10-27

**Authors:** Elisabet Berastegui Garcia, Maria Luisa Camara Rosell, Enrique Moret Ruiz, Irma Casas Garcia, Sara Badia Gamarra, Claudio Fernandez Gallego, Luis Delgado Ramis, Ignasi Julia Almill, Anna Llorens Ferrer, Bernat Romero Ferrer, Antoni Bayes Genis, Christian Muñoz Guijosa

**Affiliations:** 1 Cardiac Surgery Department, Hospital Universitari Germans Trias i Pujol, Crtra Canyet s/n, 08914 Badalona, Spain; 2 Anesthesiology Department, Hospital Universitari Germans Trias i Pujol, Badalona, Spain; 3 Preventive Medicine Department, Hospital Universitari Germans Trias i Pujol, Badalona, Spain; 4 Cardiovascular Institut, Hospital Universitari Germans Trias I Pujol, Badalona, Spain

**Keywords:** Aortic valve replacement, Frailty, Risk scales

## Abstract

**Background:**

Frailty is a geriatric syndrome that diminishes potential functional recovery after any surgical procedure. Preoperative surgical risk assessment is crucial to calibrate the risk and benefit of cardiac surgery. The aim of this study was to test usefulness of FRAIL Scale and other surgical-risk-scales and individual features of frailty in cardiac aortic valve surgery.

**Methods:**

Prospective study. From May-2014 to February-2016, we collected 200 patients who underwent aortic valve replacement, either surgically or transcatheter. At 1-year follow-up, quality of life measurements were recorded using the EQ-5D (EuroQol). Univariate and multivariate analyses correlated preoperative condition, features of frailty and predicted risk scores with mortality, morbidity and quality of life at 1 year of follow-up.

**Results:**

Mean age 78.2y, 56%male. Mean-preoperative-scores: FRAIL scale 1.5(SD 1.02), STS 2.9(SD 1.13), BI 93.8(SD 7.3), ESlog I 12.8(SD 8.5) and GS 7.3 s (SD 1.9). Morbidity at discharge, 6 m and 1 year was 51, 14 and 28%. Mortality 4%. Survival at 6 m/ 1-y was 97% / 88%. Complication-rate was higher in TAVI group due to-vascular complications. Renal dysfunction, anemia, social dependence and GS slower than 7 s were associated with morbidity. On multivariate analysis adjusted STS, BI and GS speed were statistically significant. Quality of life at 1-year follow-up adjusted for age and prosthesis type showed a significant association with STS and FRAIL scale scores.

**Conclusions:**

Frailty increases surgical risk and is associated with higher morbidity. Preoperative GS slower 7 s, and STS and FRAIL scale scores seem to be reliable predictors of quality of life at 1-year follow-up.

## Background

Aortic stenosis (AS) is one of the most common valvular diseases in developed countries. AS is closely associated with age and its prevalence is now estimated at 12% in patients older than 75 years [1–3]. Symptomatic AS, if untreated, carries a poor prognosis [2, 3]. Medical treatment is ineffective and aortic valve replacement (AVR) is still the gold standard treatment for this condition. According to the Spanish Cardiac Surgery Association Registry [4], AVR represents 42.5% of all surgical activity on an isolated valve. Currently, 45% of aortic prostheses implanted in Spain are bioprostheses due to an aging population and to avoid the increased comorbidity associated with long-term anticoagulation [4–9]. The combination of this surgical activity and the increased life expectancy in developed countries in recent decades implies a high economic cost [5]. One of the current challenges for health care services is to improve quality of life (QOL) and life expectancy in an aging population with different pathologies, such as AS [9].

In recent years, various techniques have been developed to reduce the aggression of conventional surgery in a population with an already high morbidity. In particular, transcatheter aortic valve implantation (TAVI) has shown better outcomes than medical treatment in inoperable patients [10] and non-inferiority to conventional surgery in high-risk patients [11]. Other studies such as the SURTAVI / PARTNER III trial were designed to test the effectiveness of TAVI in intermediate-risk patients. However, they did not consider other scenarios, such as treatment of AS by minimally-invasive surgery combined with the latest generation of sutureless prostheses [12].

TAVI is no longer limited to patients considered high surgical risk but is increasingly performed in those at intermediate risk; it is therefore important to determine the most reliable tools for preoperative assessment.

In this context, frailty is an increasingly important issue. Frailty is defined as an impaired physiological response to stressors, with increased vulnerability to adverse outcomes after medical or surgical stressful conditions. Its phenotype includes weight loss, tiredness, weakness and decreased physical activity. Unfortunately, although frailty is associated with an increased risk of late mortality in patients with cardiac disease, it is not currently considered in existing surgical risk models [13, 14]. Although the prevalence of frailty increases with age, age is not the only related factor.

Gait speed (GS) defined as the time taken to walk 5 m – has been shown to be correlated with frailty. GS is easy to measure and does not require a geriatric specialist. Previous studies have demonstrated a relationship between slower gait and reduced survival in older patients with heart disease [13]. This suggests that risk stratification combining current risk scales with other features such as GS could improve the prediction of morbidity and mortality in this group of patients. This may in turn have a favorable impact on the short- and long-term prognosis after surgery, especially for QOL and functional capacity [15].

## Methods

Single-center prospective cohort study involving patients older than 70 years who underwent elective aortic valve replacement, with follow-up assessment at 1 year. Between May 2014 and February 2016, 200 consecutive patients from a large public hospital were included (Hospital Germans Trias i Pujol, Badalona, Spain). All the patients signed an informed consent.

Data were collected preoperatively on variables related to cardiovascular health and frailty and postoperatively on immediate complications and follow-up. Inclusion criteria were age older than 70 years, and being scheduled to undergo elective TAVI or surgical AVR (with or without coronary procedure). Patients were excluded if the intervention was not elective, if they were in cardiogenic shock, had a diagnosis of endocarditis, were undergoing a redo procedure or a procedure involving ascending aortic surgery or any other concomitant procedure, if unable to attend a 1-year follow-up assessment, or if they refused to provide informed consent.

Postoperative evaluation was performed at the time of hospital discharge and at follow-up. The primary end points were all-cause mortality and in-hospital morbidity. Morbidity is defined as respiratory (intubation / tracheostomy, etc.), cardiovascular (pacemaker), infectious or renal complications, longer stay in ICU or ward (more than 2 days or 7 days).

At discharge, patients were scheduled for follow-up at 30 days, 6 months and 1 year, with clinical assessment, echocardiogram and QOL assessment with the EuroQol Group’s EQ-5D questionnaire. Follow-up was completed through to the last follow-up visit for all living participants.

### Baseline frailty assessment

Patient frailty was assessed using the FRAIL scale, a simple and reliable instrument that measures patients’ preoperative frailty on the basis of 5 factors: Fatigue, Resistance, Ambulation, Illness and Loss of weight. Patients scoring under 3 on the scale are categorized as *not frail* and those scoring 3 or above, as *frail*.

Previous studies have measured the 5 components of the FRAIL scale in various ways. In our study, we measured resistance using the Barthel Index for activities of daily living. Published by Mahoney and Barthel in 1965, the Barthel Index is the instrument recommended by the British Gerontology Society to evaluate the ability of elderly patients to independently perform basic activities of daily living. It yields a score between 0 and 100. The preoperative nutritional status of patients was evaluated using serum albumin level and body muscle-mass index. Strength was measured using a hand grip dynamometer. Fatigue and ambulation were measured with a 5-m gait-speed test. All measures for frailty were taken by a single therapist.

### Surgical treatment

The prostheses used in this study included:

- TAVI: In these cases it is a trivalve biological prosthesis, of porcine pericardium, mounted and sutured on a self-expanding nitinol structure.
- Stented- biological: Bovine pericardial tissue. They are recommended in patients who do not wish to take anticoagulants, since they have very little tendency to form embolisms or thrombosis even without anticoagulants.
- Sutureless: Sutureless prostheses are composed of a nitinol structure and a biological prosthesis of bovine pericardium. In our series, it is a bioprosthesis composed of a bovine pericardium valve mounted on a NITINOL self-expanding double ring stent; and a vertical structure covered with Carbofilm. The malleable properties of nitinol makes the stent adapt to the anatomy of the root aorta reducing the stress load on the leaflets.

### Postoperative assessment

Postoperative evaluation was performed at the time of hospital discharge and at follow-up. The primary end points were all-cause mortality and in-hospital morbidity defined by length of hospital stay, respiratory, renal, vascular or neurological complications; and QOL at 1-year follow-up was a secondary end point. The number and causes of death or postoperative re hospitalizations prior to follow-up were obtained from clinical records stored in public health systems shared database.

QOL was assessed at baseline and 1-year follow-up using the Spanish version of the widely-used EQ-5D, which has been prospectively validated in Spain. The EQ-5D consists of questions covering 5 areas, namely mobility, self-care, usual activities, pain/discomfort and anxiety/depression, each yielding a score from 1 (no limitation) to 3 (extreme limitation), and a visual analog scale, with 0 indicating “the worst health you can imagine” and 100 “the best health you can imagine”.

All patients gave consent to participate, and the study was approved by the hospital’s institutional ethics committee in accordance with accepted ethical standards.

### Statistical analyses

Categorical variables were expressed as frequencies and percentages. Continuous variables were described as means ± SD or as medians and 25th and 75th percentils. Chi-square, Fisher or ANOVA tests were used to compare preoperative, intraoperative and postoperative patient characteristics; a separate analysis was performed for each treatment group (prosthesis type). QOL was analyzed using ANOVA or the Kruskal-Wallis test, also separately for each prosthesis type. The multivariate analysis of morbidity and mortality risk factors was performed using a multivariate binary logistic regression model with the statistically significant variable detected in the univariate analysis. The level of significance was set at *p* < 0.5 required to claim significance. Final validation of the model was carried out through discrimination and calibration with ROC curves. ROC curves were used to evaluate frailty scores, their relation to other risk scores and their predictive capacity for morbidity. Curves for the different prosthesis groups were compared using a log-rank test. All statistical analyses were performed with SPSS 15 (SPSS Inc.).

## Results

The average age was 75 years for all treatment groups. Only 8 patients received a concomitant coronary procedure.

Breakdown according to the type of prosthesis was 22 TAVI, 80 stented valves and 98 sutureless valves. 92.5% of patients had hypertension, 31% had diabetes, 64% had dyslipidemia and 32% had chronic obstructive pulmonary disease (COPD). Up to 30% of the patients had a diagnosis of peripheral arterial disease at the time of surgery. The TAVI group had a higher percentage of preoperative renal insufficiency, social dependence, pre-procedure hospital admissions, anemia and hypoalbuminemia (Table 1).

**Table 1:** Preoperative features

Preoperative features	Stent ED % (n) 40% (80)	Sutureless %(n) 49% (98)	Tavi %(n) 11% (22)	Total 200	p
**Age (SD)**	76.6 (3.9)	78.5 (3.9)	79.8(7.0)	78.25(4.6)	.08
**BMI (SD)**	28.1(3.6)	28.6(4.6)	30.4 (8,0)	28.65(4.8)	.07
**Gender(Male/Female)**	65%(52) 35%(28)	46%(45) 54%(53)	68%(15) 32(7)	112 (56) 88 (44)	.06
**HTA % (n)**	97.5%(78)	89.8% (88)	86.4%(19)	92.5 (185)	.07
**DM % (n).** **(ADOS/Insulina)**	32.5 (26)	19.4 (19)	77.3% (17)	31% (62)	.08
**DLP % (n)**	67.5%(54)	58.4% (57)	77.3%(17)	64%(128)	.1
**Arteriopatía % (n)**	20%(16)	30.6% (30)	63.6%(14)	30% (60)	.9
**Liver Disease %(n)**	1.3%(1)	2%(2)	4,5% (1)	4%(2)	.8
**COPD %(n)**	27.5%(22)	29.6% (29)	59.1%(13)	32% (64)	.8
**CRF %(n)**	12.5%(10)	16.3% (16)	27.3%(6)	16% (32)	.6
**Social dependence**	6.3%(5)	17.3% (17)	59.1%(13)	17.5%(35)	.06
**Preop. Income**	51.2%(41)	51%(50)	95.5%(21)	56% (112)	.07
**Anemia < 34%**	23.8%(19)	19.4% (19)	72% (16)	27% (54)	.06
**Albúmin < 34 g/l**	8.8% (7)	8.2%(8)	31.8%(7)	11% (22)	.08
**Depression treatment %(n)**	6.3%(5)	12.2% (12)	9.1% (2)	9.5% (19)	.09

*SD* Standard Desviation

*BMI* Body mass index HTA High blood pressure, *DM* Diabetes mellitus (ADOs oral treatment), *DLP* Dyslipidemia, *COPD* (Chronic obstructive pulmonar disease), enfermedad pulmonar obstructiva crónica), *CRF* Chronic renal failure

T-Student. p .05

Concerning features of frailty, the TAVI and sutureless groups obtained higher scores on the risk scales and worse scores for dependency or Barthel Index than the stented group. The results of the GS and strength test (handgrip) are shown in Table 2.

**Table 2:**
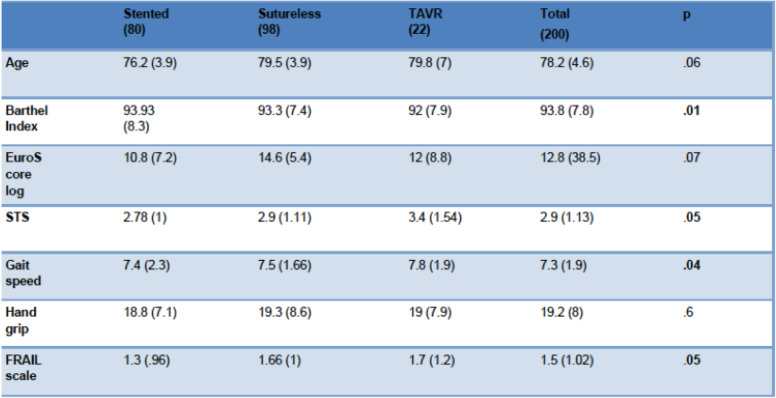
Frailty features and risk scores

*STS* Society of Thoracic Surgeons score

^1^T Student Fisher. p.05

The intraoperative characteristics are shown in Table 3.

**Table 3:**
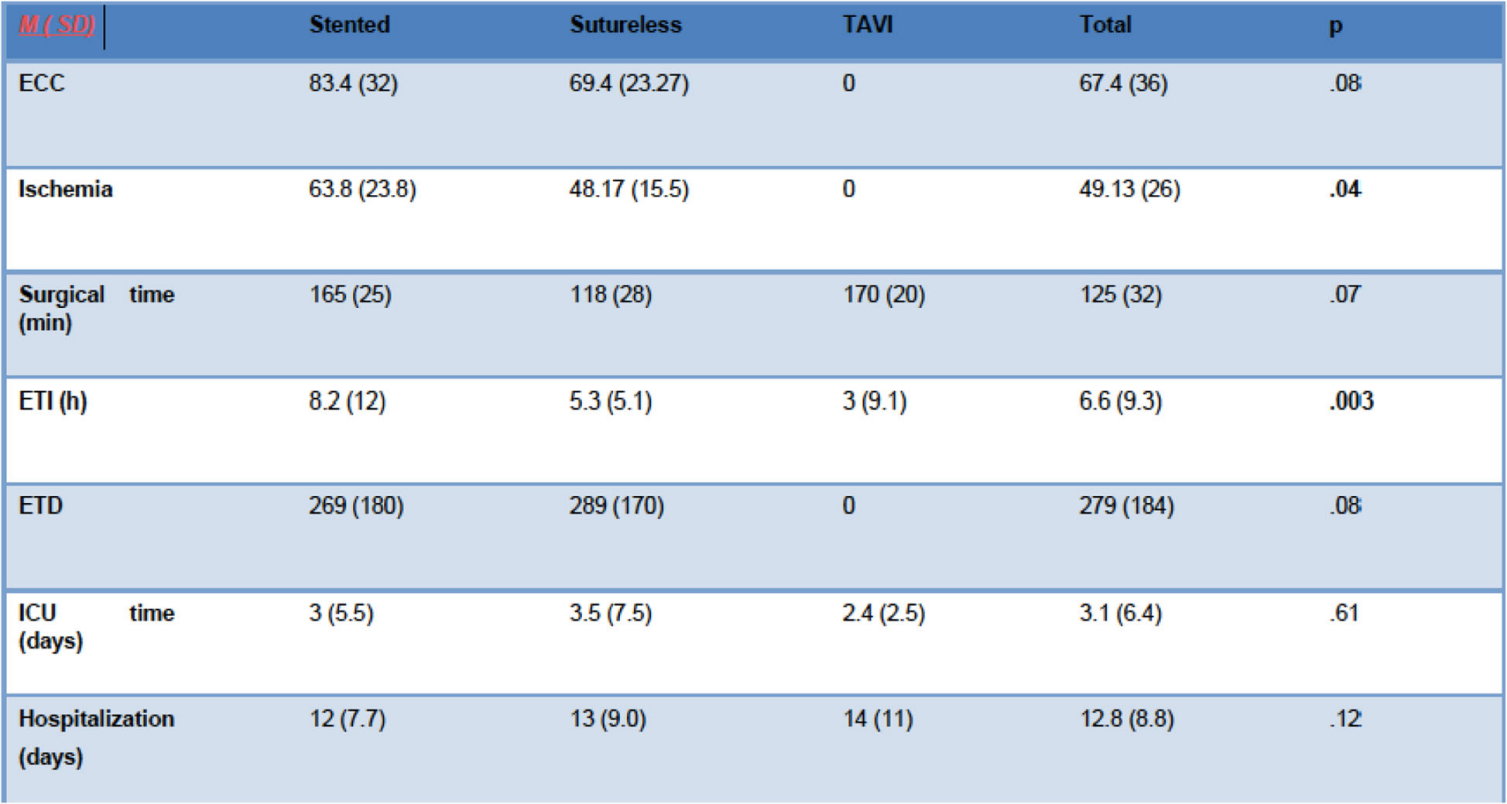
Surgical data

*ECC* Extracorporeal circulation time, *ETD* Endothoracic drainage, *ETI* Endotracheal intubation (oral), *h* Hours, *ICU* Intensive care unit, *min* Minutes, *TAVR* Transcatheter aortic valve replacement

Only the stented and sutureless groups were comparable in terms of use of extracorporeal circulation. For both these groups, the rate of immediate complications was close 43% - defined as the occurrence of any respiratory event, prolonged intubation, infectious or vascular complication, deterioration of renal function or neurological event (Table 4).

**Table 4:**
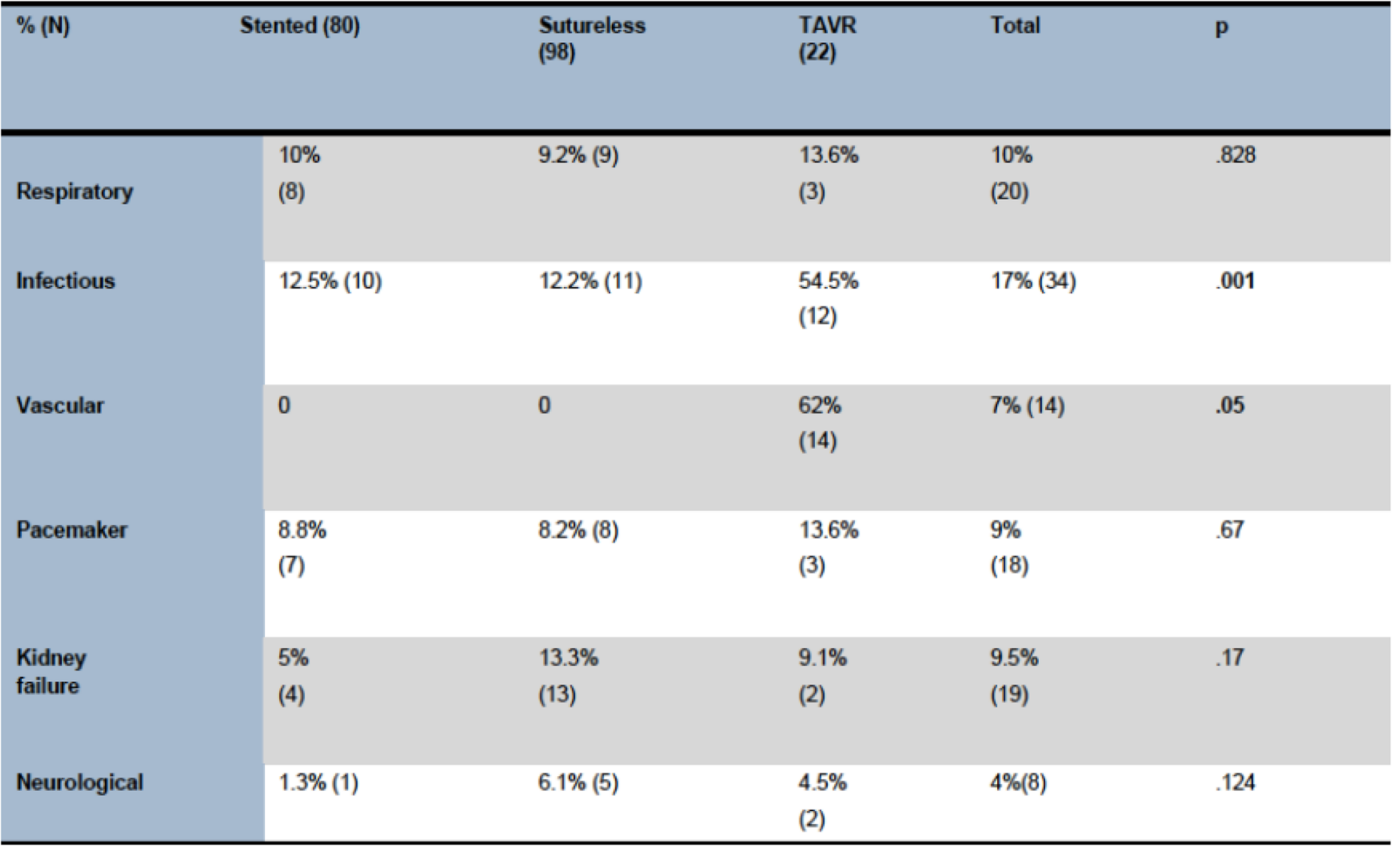
Postoperative complications

By follow-up at 1 year, the occurrence of complications and re-admissions was at 14–29% respectively the most frequent complications being respiratory or exacerbation of renal disease or heart failure. The TAVI group had more vascular and neurological complications than SAVR groups.

**Fig. 1:**
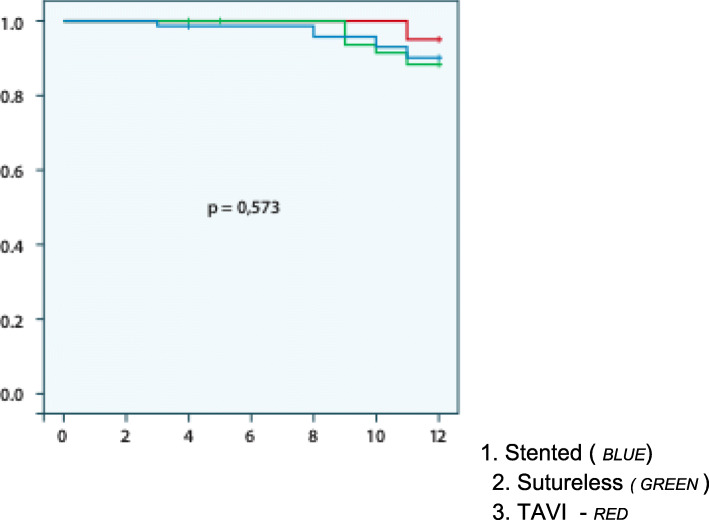
Kaplan Meier

Mortality was 4% (8 patients) at discharge, 2.6% at 6 months and 12.6% at 1 year, with no differences between groups (Fig. 1). The main cause of death in the follow-up period was progression of chronic disease (heart failure and pulmonary disease) followed by oncological disease. 2.6% of patients were diagnosed with a de novo oncological process, with 80% mortality at 1-year follow-up in this group.

In the univariate analysis, only preoperative anemia, arterial disease, renal insufficiency, GS > 7 s, Barthel Index and STS score were significant predictors of morbidity. Adjusted for prosthesis type and age, in the multivariate analysis, these were reduced to only Barthel Index, GS > 7 s, and STS score. Adjusted for patient characteristics, only gait speed, FRAIL score and STS score showed a significant relationship between preoperative values and improvement in QOL at 1-year follow-up (Tables 5 and 6).

**Table 5:**
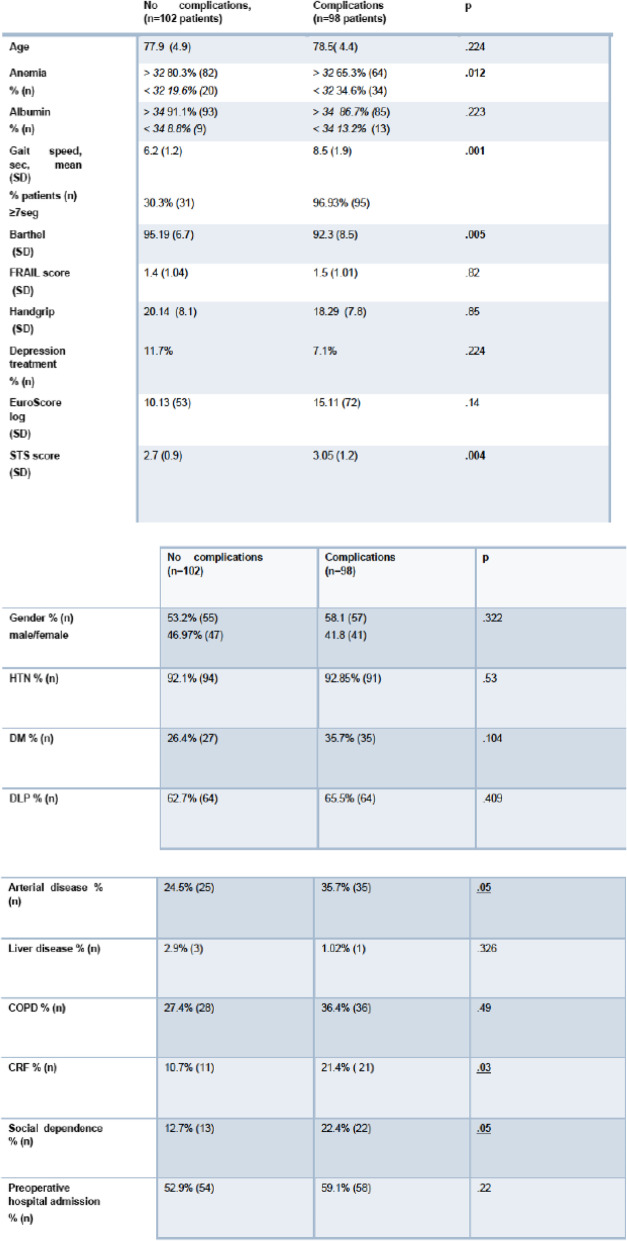
Univariate Analysis

*COPD* Chronic obstructive pulmonary disease, *CRF* Chronic renal failure, *DM* Diabetes mellitus, *DLP* Dyslipidemia, *HTN* Hypertension

Univariate. T. Student p.05

**Table 6:**
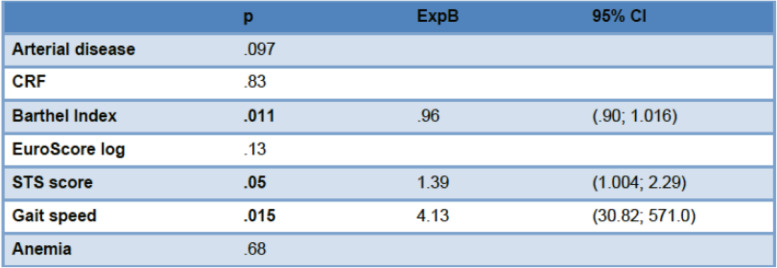
Multivariate analysis. Factors predictive of morbidity in cardiac surgery

CRF: chronic renal failure; STS: Society of Thoracic Surgeons

Logistic Regression Multivariate

**Fig. 2:**
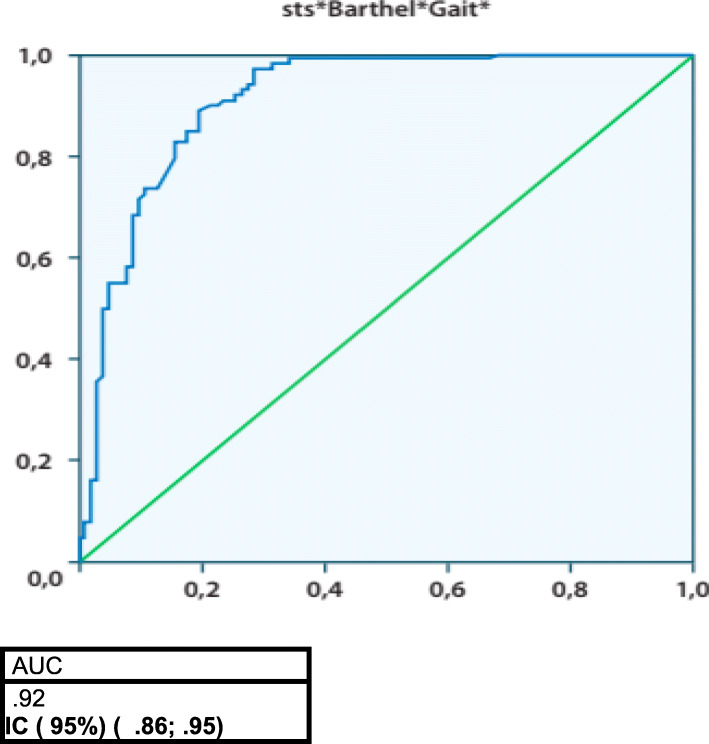
ROC - STS / Barthel / Gait - morbidity

An ROC study showed that the multivariate analysis with morbidity was superior to the univariate analysis for any individual feature and morbidity (Fig. 2).

## Discussion

AS is the third most frequent cardiovascular disease in the Western world, where its prevalence is around 18% in the octogenarian population [1, 2]. Aortic valve replacement is currently the only definitive treatment for patients with AS, showing good long-term outcomes even for populations at high and intermediate risk [16, 17]. Preoperative estimation of surgical risk is essential during the therapeutic decision-making process. Current risk models are based on demographic and clinical factors, and other measurable factors of comorbidity, but do not include a comprehensive assessment of frailty, which is known to affect morbidity and mortality.

The potential impact of a procedure on a patient’s long-term QOL must also be considered when selecting one surgical procedure over another. Preoperative measures of frailty appear to be predictive of postoperative QOL [18–20].

In the present prospective study, 200 patients undergoing aortic valve replacement were divided into 3 groups according to AVR surgical procedure. The study population was homogenous in terms of preoperative predictors of cardiovascular risk, but the TAVI group showed higher preoperative rates of peripheral arterial disease, kidney disease, hypoalbuminemia, anemia and preoperative admission rate. The sutureless and TAVI groups had higher scores for risk (STS) and frailty (FRAIL scale) than the stented prosthesis group. Overall survival at discharge, 6 months and 1 year was 96, 97 and 89% respectively. At 1-year follow-up 26 patients had died.

Our results on preoperative features are similar to those reported in the European Cardiac Surgery Registry for stented/sutureless prosthesis implantation surgery in intermediate- to high-risk populations [21] and in the Spanish Cardiac Surgery Registry for TAVI. Mortality rates at 6 months and 1 year were 2.6 and 11%, respectively, similar to other previously published series. However, the 4% 30-day mortality, was lower than that reported in various TAVI registries (Spanish, Canadian, French, British, Italian and German), which ranges between 8 and 10% and 16–24%/year [4, 21–24].

The rate of postoperative complications was highest in the TAVI group, when compared to the SAVR group and higher than that described in other TAVI registries. This was mainly due to vascular and infectious complications, which may have been related to the high rate of peripheral vascular disease and transfemoral approach.

Complications during the follow-up period were related to the patients’ preoperative condition and comorbidities. Respiratory complications or cardiac failure occurred in over 8% of patients at 6 months and 18.2 to 23% at 1 year. The leading cause of death was progression of respiratory or renal disease, and the second was oncological disease.

### Preoperative factors related to morbidity and mortality

As in other studies, our univariate analysis found that arterial disease, anemia, STS score, Barthel Index and gait speed were associated with morbidity. After adjusting for age, only STS score, Barthel Index and gait speed were significant. Aging generally involves a decrease in muscular strength, related to loss of muscle mass and protein reduction, a factor measured by handgrip, although no association with morbidity was found [13, 14, 25, 26].

GS is an easily measured patient feature and has been shown to correlate with complications in patients with heart failure and cardiac surgery [13]. It is a useful tool for predicting morbidity in elderly patients and has been shown to be related to resilience. This association not only has clinical implications, but also has a bearing on long-term quality of life. In addition, there is also an overall economic impact to consider, which should take into account not only direct costs but also indirect costs incurred due to intraoperative and postoperative complications.

To date, gait speed has been used as an isolated parameter. In our study, gait speed combined with STS and Barthel score increased its predictive capacity for morbidity.

### Quality of life at follow-up

Frailty increases surgical risk. Some studies have found that even with less invasive techniques, QOL outcomes are not necessarily improved in intermediate- to high-risk patients. One of the most widely-used and validated scales for measuring QOL in our setting is the EQ-5D which assesses patients’ physical and psychological condition [27, 28]. In contrast to what is reported in other studies, the TAVI group in our study had a worse QOL at 1 year than those who had received a conventional or sutureless prosthesis. Furthermore, as in other studies, TAVI patients also reported a worse QOL in items related to their affective or psychological wellbeing, not just in the functional–motor sphere [29].

Some previous studies have found an association between preoperative factors such as peripheral arterial disease and mood and worse QOL. Nevertheless, there are no studies that assess the association between preoperative risk factors or frailty features with QOL at follow-up. In our univariate analysis, faster gait speed and higher STS and FRAIL scale scores were negatively correlated with QOL at 1-year follow-up, but in multivariate analysis of these 3 factors only STS and FRAIL score remained predictors of worse QOL [30].

Thus, regardless of the surgical procedure employed, only STS and FRAIL score were strong predictors of QOL at 1-year follow-up. The importance of this conclusion becomes evident on analysis of the individual prosthesis types: although in our study there were relatively few TAVI interventions compared with stented or sutureless valve replacements, TAVI did not appear to be associated with improved QOL. It is therefore important to emphasize that the use of risk and frailty scales improves the prediction of morbidity and, most importantly, quality of life long term.

## Conclusions

The combination of STS score, Barthel Index and gait speed appears to have considerable predictive power for morbidity. In addition, we have shown that preoperative frailty assessment is a useful predictor of patient quality of life at 1-year follow-up. It is important to know which patients will benefit from which types of prosthesis to reduce immediate morbidity and to improve clinicians’ confidence regarding the quality of life that we can afford our patients.

## Data Availability

**• Right to privacy and informed consent.** The authors have obtained the informed consent of the patients and / or subjects referred to in the article. • The datasets generated and/or analysed during the current study are available in the Tesis Doctorals en XArxa (TDX) repository, http://hdl.handle.net/10803/461594.

## Abbreviations

AS: Aortic stenosis
AVR: Aortic Valve replacement
BI: Index Barthel
COPD: Chronic Obstructive pulmonary Disease
EQ 5D: Euro Quol Test
GS: Gait Speed
QuOl: Quality of life
STS: Society Thoracic Surgeons
TAVI: Transcatheter Aortic valve implantation
